# The Children of the Rich

**Published:** 1854-06

**Authors:** 


### THE CHILDREN OF THE RICH.

The following article ought not to perish with a daily paper.

" Too much honor cannot be awarded to Messrs. Brace and Pease for their untiring efforts to elevate the children of the city poor, from the conditions of ignorance and general demoralization in which so many of them lie enthralled. We every day hear interesting narratives of the good which is doing by these instrumentalities, of intelligence quickened, virtuous resolutions enforced, industrial aspirations promoted; and we are told that the testimony of the farmers in the country, upon whose wholesome stock these youthful scions are sought to be engrafted, is often extremely encouraging. Messrs. Pease and Brace are ndeed sure to be rewarded in the advancing success of their enterprise.

" But we are satisfied that there is a still more hopeless class among us than the children of the Five Points, and these are the children of our rich men. The former have this advantage, that they are born and nurtured under circumstances of so much infamy, as to make any change in their condition almost necessarily a change for the better and not for the worse. They begin at the very lowest step of the social ladder, and although they may in truth never mount, they yet may hardly be said ever to descend any lower than their original perch. No affable pimp is so foolish as to lavish his attentions upon the outcast and penniless, nor does the unctuous blackleg deem it worth while to lubricate by the fatal saliva of *his* courtesies, a morsel which when swallowed must prove so purely sinewy and undigestible. Thus the baseness of our Five Points children is apt to remain native, not acquired. They have any amount of "original sin" on hand, but their "actual transgressions" pale and die out before the lurid glow which characterizes those of our Fifth Avenue youth.

"Where then is the benevolent Mr. Pease or Mr. Brace whose heart is touched by the moral raggedness of our rich young men? Where is the bold and wise philanthropist who shall probe this deadly and deepening ulcer, and tell us what soundness remains underneath? The time is ripe, the urgency unprecedented. One can count as he goes along our lordly thoroughfares, *so many* homes in which the father sits solitary, robbed of the sons who should have been the ornament and prop of his declining years, or in which the sleepless heart of the mother counts the weary hours till morning, waiting in vain her prodigal's return! And one can also count on the other hand as he goes along Broadway *so many* princely houses where hell lies in ambush, and hecatombs of promising youth are nightly offered up to the gigantic Moloch of Play! We are informed on good authority that fifteen houses between Bleecker and Barclay-streets in Broadway alone, are daily and nightly open for gambling, fitted up many of them with extreme luxury, rendered attractive by every artifice which can inflame the senses and captivate an imagination devoted to pleasure, and maintained some of them as to the mere necessary expenditures at an outlay of between twenty and thirty thousand dollars a year. Who support these glittering palaces of death? Where, for instance, do the proprietors of the most luxurious of these hells get the twenty or thirty thousand dollars *per annum*, which enable them to maintain the house they occupy between Prince street and Spring, and set a dinner table and a supper table every day and night, which eclipse every gentleman's table in town, and which, nevertheless, are *free* to every gentleman's son in town? They get them out of the pockets of our business men. The industry and enterprise of our commercial classes are incessantly tapped to fatten these bloated ulcers of vice and crime. For it is not the sons of our farmers and mechanics that are to be found in these haunts. but only the sons of those who have large property, and expect to leave their children enough to maintain them without work. It is the children of our rich men who keep up the army of pimps, and swindlers, and black-legs that infest the city. Find a young man who has no money, or who having money, has no desire to get rid of it unprofitably, and you find a soil upon which roguery cannot fasten. Who feed our pugilists? Who in the long run pay the expenses of their idleness, and train them for their loathsome office? It is of course our rich men. It is those who having amassed a mint of money, carelessly and culpably drown the active or productive energies of their children in the love of purely passive enjoyment.

The mud of our streets owes half its parentage to the dust of the earth, and half to the rains of heaven. So the vice and crime which disfigure society appear to grow out of the alliance of extreme wealth and extreme poverty. It is chiefly in the very lowest or in the very highest stages of the social edifice that we encounter intemperance, licentiousness, gambling, and the various forms of profligacy which still curse our civilization. We have all faith, indeed, that as Henry C. Carey, and after him, Frederick Bastiat, have splendidly demonstrated, there exists a perpetual tendency in history toward the approximation of our social extremes, by the gradual elevation of both to a new social level; but in the meantime how desirable would it be to have this faith intelligently promoted by our own action! How much, meantime, might our rich men do by cutting off as far as in them lies, the sources of the existing demoralization! As the reader passes along Broadway, let him glance at the juvenile faces that about noon-day fill the porches and sitting-room windows of the great hotels, and if he be a father let him ask himself how he would like to see a son of his own enrolled in that bleached and decrepid regiment. How still they sit, and how patiently they gaze upon the monotonous streets! Are they palsied? No, they smoke, they sneeze, they cough, they discharge in fact all the offices of automatic life. By-and-by they will rise and saunter toward the bar perhaps, or they will go to the billiard-room and chase the weary hours around the table till dinner-time, when night will doubtless galvanize them into some more feverish activity. You devoutly pray God to exempt your darling boy from such a fate as he grows up. But God's pity is infinite toward these poor faded flowers, and your blooming offspring can claim no exceptional regard from him. By no coaxing or adulation can we persuade Him to remit eternal laws in our behalf, and if we bring up our children to covet a life of pleasure as the *summum bonum*, or to anticipate a career of inglorious or passive enjoyment, not all the powers of heaven can prevent their falling into the hands of the harpies who live by their destruction. Of course it is entirely right that the enterprise of our business men should be richly rewarded, that industry and fidelity to one's avocations should even be stimulated by the chance of attaining at last to abounding wealth. But at the same time let us all remember *that we belong to society before we belong to ourselves*, and that we have no right therefore to overlook the paramount claims of society for a moment in the education of our children. The grand distinction of human life is that it is pervaded by the sentiment of society, fellowship, equality, and those accordingly in all ages who have most amply illustrated it have been marked by the most cordial subjection to this sentiment. We would have fathers remember that their children *are primarily the children of society*, and only secondarily theirs. And we repeat that they have no right to overlook the paramount claims of society in the education of their children. No man, even supposing him to have the wealth of Mr. Astor, has a right to bring up his children to a career of idleness. No man not a savage has a right to educate his children with a view simply to the passive enjoyment of life. This is wholly to mistake the end and meaning of life. Life was never meant to be a mere pleasure save to the brute. To higher natures it has always been and always will be a school, a discipline, a journey, a march, a battle, a victory. The law is absolute and wholesome, growing out of the very divinity of man's source. No amount of fortune accordingly can exempt a man from its operation. It leaves no one where it finds him. If it does not elevate him above the lambent stars, it makes him grovel in the dust of the earth. The alternative is infallible, and therefore we say to our thoughtless rich men, that they had better, on every account study the methods of a wise depletion, and educate their children to industry, economy, usefulness. It were greatly better for society, because society would then have immense benefits unsparingly rendered it; and it were greatly better also for their sons, because then these latter would stand some chance of turning out the men their fathers were before them, and would no longer be tempted to curse the parentage, whose fond and wicked pride furnished them the means only of a boundless and inevitable profligacy."—*New York Tribune*.

